# Comprehensive review of Korean Medicine registries 2015–2023

**DOI:** 10.3389/fmed.2024.1412053

**Published:** 2024-09-18

**Authors:** Soo-Dam Kim, Sunmi Choi, Sungha Kim

**Affiliations:** ^1^KM Science Research Division, Korea Institute of Oriental Medicine, Daejeon, Republic of Korea; ^2^KM Data Division, Korea Institute of Oriental Medicine, Daejeon, Republic of Korea; ^3^Korean Convergence Medical Science, KIOM School, University of Science and Technology (UST), Daejeon, Republic of Korea

**Keywords:** Korean Medicine, registry, evidence-based, review, research trends

## Abstract

**Background:**

Despite the increasing popularity of Korean Medicine (KM), its scientific evidence faces scrutiny. Instead of randomized controlled trials, registries are favored to capture the real world of KM practice due to the difficulties associated with proper control and the holistic nature of the KM approach. This review aimed to examine the KM registries in detail, identify the scope and focus of studies within this field, and assess the research trends.

**Methods:**

We conducted a comprehensive analysis of KM registries listed in trial registration platforms, covering records from their inception until the end of 2023. The selection criteria aimed to include studies focusing on various interventions related to KM, with data extraction focusing on study characteristics and outcomes measured. The analysis utilized descriptive statistics to summarize the findings.

**Results:**

We identified a steady increase in registry studies (2015, one; 2023, seven). Musculoskeletal disorders were most studied (28%), aligning with patients’ demand. The involvement of 112 primary clinics and Quality of Life (QOL) as the predominant outcome in 14 (66.7%) registries demonstrates the positive impact on patient well-being and the critical role that primary clinics play in KM practice.

**Conclusion:**

Our findings indicate a heightened interest and commitment to evidence-based KM practices. Future Registries should be implemented on a large scale, incorporating long-term follow-up encompassing primary clinics. This approach would enable a comprehensive evaluation of the effectiveness and safety of KM interventions, as well as offer valuable insights into the influence of KM on chronic conditions and QOL.

## 1 Introduction

Korean Medicine (KM), which is an integral part of East Asian traditional medicine, is characterized by a holistic approach that focuses on balancing the vital energy of the entire body (1). It encompasses a range of practices, including herbal medicine, acupuncture, and mind–body therapies. Formed under the influence of ancient Chinese medicine, KM has evolved in tandem with Traditional Chinese Medicine, with both systems influencing each other over centuries (2). However, unlike Chinese medicine facilities, most KM facilities are primary clinics, which impedes the conduct of large-scale controlled clinical trials (3) due to limited personnel and resources.

Accordingly, most related studies have been small-scale studies, such as case series, which may not offer the comprehensive data required for scientific validation (3). Moreover, randomized controlled trials (RCTs) may not be ideally suited to evaluate the holistic and individualized characteristics of KM modalities (4, 5) since they involve complex, tailored treatments aimed primarily at restoring body balance. This focus on the restoration of homeostasis, dynamics, and vitality approaches the individual as an integrated whole, rather than merely targeting isolated pathological entities (6). Consequently, there is a need to establish new research methodologies that align with KM characteristics and allow assessment of the clinical effectiveness and safety of KM as well as facilitate the exploration of KM theories through large-scale data analysis.

Clinical registries are structured systems used to collect, manage, and analyze health-related data for a specific population, condition, or treatment (7, 8). These registries are designed to systematically gather detailed medical information from patients over time, providing a rich resource for understanding disease progression, treatment outcomes, and patient safety (9). Unlike RCTs, clinical registries capture data in real-world settings, offering insights into how treatments perform in diverse and everyday clinical scenarios (10). These data can include patient demographics, diagnostic information, details of treatments received, and outcomes of those treatments (2020) (9). By harnessing the power of large datasets, clinical registries facilitate observational studies and post-marketing studies, which contributes toward elucidation of healthcare interventions (11). This approach is particularly suited for KM settings where large-scale RCTs are less feasible.

Accordingly, this study aimed to explore the current status of clinical KM registries. Specifically, this study aimed to analyze information from ClinicalTrials.gov, the International Clinical Trials Registry Platform (ICTRP), and the Clinical Research Information Service (CRIS) in order to gain insights into the methodologies and demographics involved in KM research. Our objective was to review the past registration status of Korean Medicine in registries in order to guide future research.

## 2 Methods

### 2.1 Database sources

We comprehensively utilized KM registry information from the ClinicalTrials.gov, ICTRP, and CRIS, covering the period from their inception to the end of 2023. These databases were selected due to their comprehensive coverage of clinical studies in KM, which yielded a broad and representative sample of KM research.

### 2.2 Data collection and selection criteria

This study included registry studies focusing on KM interventions such as herbal medicine, acupuncture (including electroacupuncture, pharmacopuncture, and thread embedding), moxibustion, massage, and mind–body therapies. Searches were conducted in both Korean and English, using keywords like Korean Medicine, herbal medicine, acupuncture, registry, and Korea, in order to capture a diverse collection of KM practices. Our inclusive approach did not restrict disease conditions, age, or sex of participants. We excluded duplicate studies across the databases, non-registry-based studies, and studies conducted outside South Korea in order to focus on native KM practices. The analysis did not impose a predefined definition for endpoint outcomes, which allowed the inclusion of a wide array of outcome measures reported in KM studies.

### 2.3 Data extraction

We utilized a data extraction form to systematically gather essential information from each included KM registry such as study registration number, registration and ethics approval dates, public and scientific titles, sponsor organization and study site, study status, primary sponsor, study design, actual start and completion dates, sample size, intervention measures, inclusion and exclusion criteria, participant demographics (age, sex, health condition), and primary and secondary outcomes. Missing data were supplemented from study descriptions or marked as Not Reported (N/R) when unavailable.

### 2.4 Analysis methodology

For the analysis of coded data within our study, we employed Microsoft Excel (Version 2108), leveraging its capabilities to systematically organize and assess the collected information. We focused on categorical data, which are expressed as absolute numbers (*n*) and relative percentages (%).

## 3 Results

### 3.1 Search results

**Figure 1:**
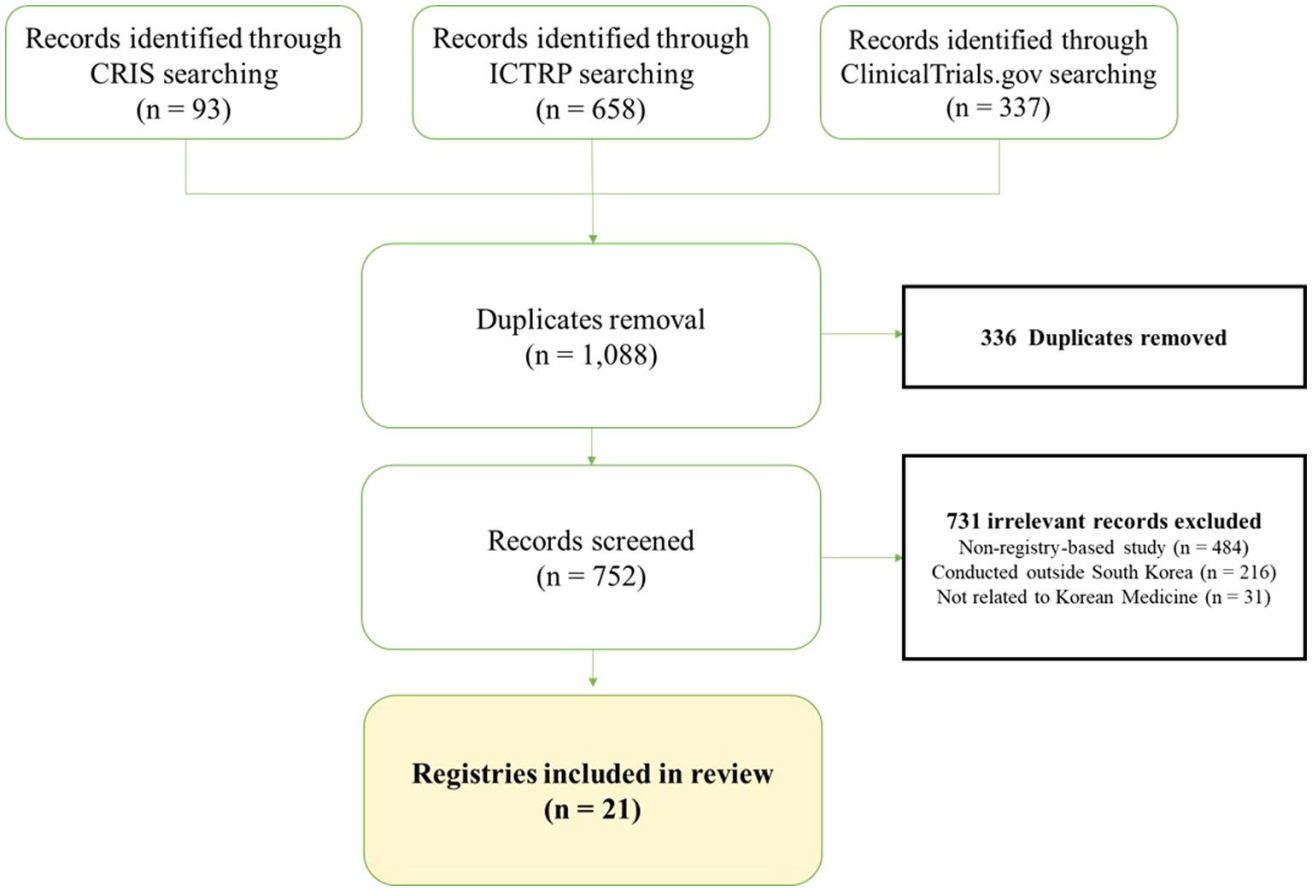
PRISMA flowchart of the selection process for Korean Medicine registries.

Figure 1 shows the publication retrieval process. From an initial 1,088 identified records, 336 were removed due to duplication and 731 were excluded for not being relevant to KM registries, including 484 non-registry-based studies, 216 studies conducted outside South Korea, and 31 studies not related to Korean Medicine. Consequently, we selected 21 eligible studies on KM registries. Supplementary Table S1 provides the detailed characteristics of the selected KM registry studies.

### 3.2 Registration trends of the KM registries

**Figure 2:**
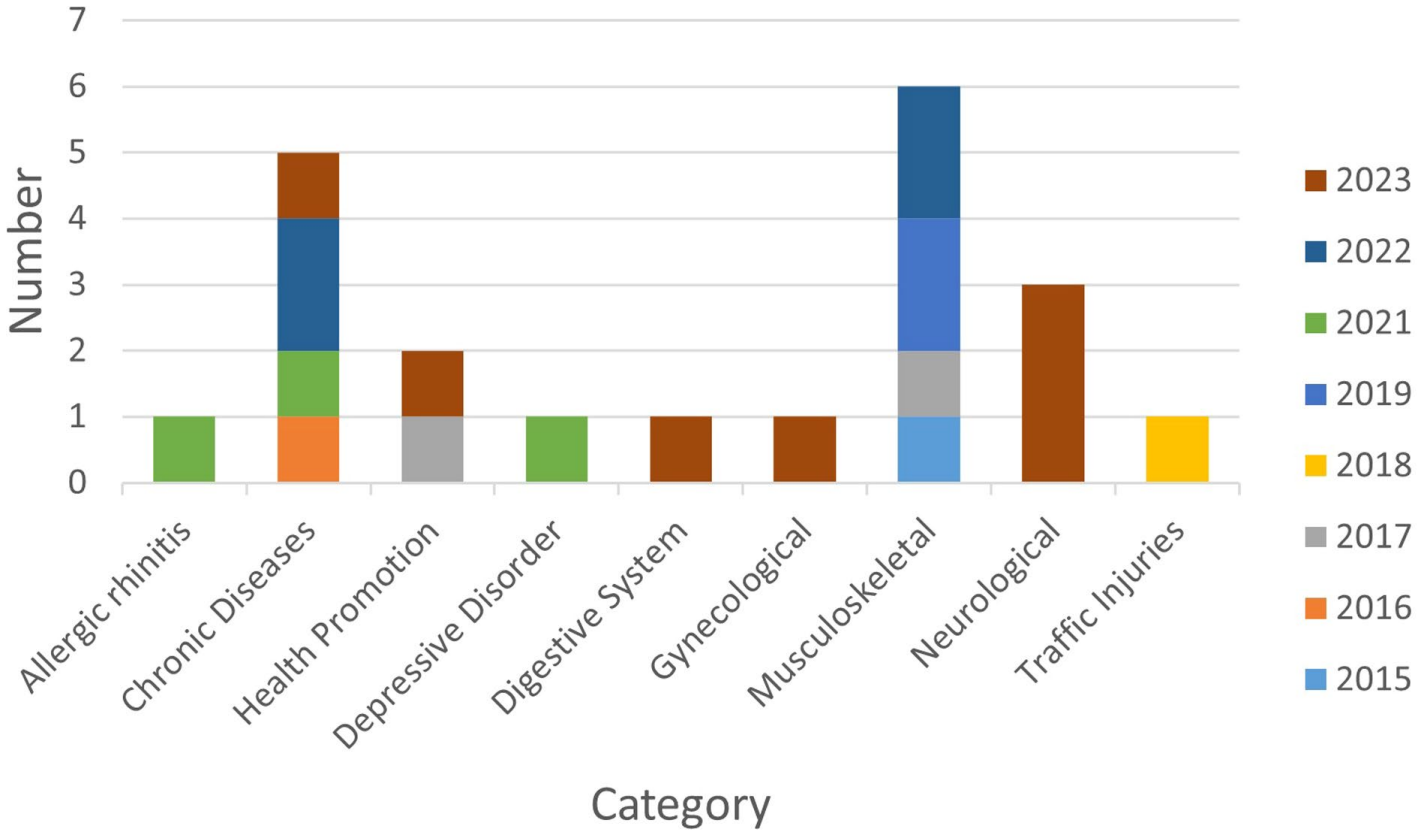
Categorization and annual distribution of Korean Medicine registries.

The included KM registries were registered between 2015 and 2023, with a trend of a steady increase from one in 2015 to seven in 2023 (Figure 2). The first registry study, which was registered on March 30, 2015, was an observational multicenter study trial titled “Korean medicine registry for low back pain-prospective observational multicenter study” (KCT0001427, NCT02418286), and it was sponsored by Gil Korean Medicine Hospital, Gachon University. The organization that conducted the highest number of registry studies during this period was Kyung Hee University (*n* = 7), followed by Korea Institute of Oriental Medicine (*n* = 3) and Pusan National University (*n* = 3).

### 3.3 Prevalence of conditions in KM registries

Musculoskeletal disorders were the most studied condition with six registries (28%), followed by chronic diseases with five registries (24%). Regarding chronic diseases, neoplasms and obesities were studied in two registries (9%) each, while essential hypertension was studied in one (5%). Neurological conditions were studied in three registries (14%), including two studies (9%) on stroke and one (5%) on facial palsy. Two (9%) registries focused on health promotion, while there was one (5%) registry each on the respiratory disorders, gynecological disorders, depressive disorder, traffic injuries, and digestive disorders (Figure 2).

### 3.4 Characteristics of the KM registries

Ethics approval was granted in 95.2% of KM registries, with the remaining registries having an unclear status. Hospitals were the predominant sponsor organizations (61.9%), followed by universities (19.0%), research institutions (14.3%), and a primary clinic (4.8%). Registries typically focused on specific diseases or conditions (52.4%), treatment methods (28.6%), and patient groups (19.0%). The sample sizes varied, with most studies (33.3%) including 100–199 participants. Large cohort studies with 200–499 and ≥ 1,000 participants were equally represented (23.8% each), while smaller studies with <100 and 500–999 participants were less frequent (9.5% each). Completed studies comprised 66.7% of the registries, with the remaining registries (33.3%) still recruiting. Most studies were multi-center, with 52.4 and 28.6% of the studies involving <10 and ≥ 10 centers, respectively, highlighting a preference for collaborative research efforts across sites. Single-center studies accounted for 19.0% of the registries. Among the participating institutions, there were 112 primary clinics and 68 hospitals, indicating the active participation of various healthcare settings in advancing KM research. Among the 21 included registries, 10 published their protocol, with only one registry (KCT0006625) publishing both the protocol and the results (12, 13) (Table 1; Supplementary Table S1).

**Table 1:** Characteristics of the KM registries.

Item	Detail	Record [*n* (%)]
Ethics approval	Yes	20 (95.2)
	Unclear	1 (4.8)
Sponsor organization	Hospital	13 (61.9)
	University	4 (19.0)
	Research institution	3 (14.3)
	Primary clinic	1 (4.8)
Types of registries	Disease/Condition	16 (76.2)
	Treatment method	8 (38.1)
	Patient group	5 (23.8)
Sample size	< 100	2 (9.5)
	100–199	7 (33.3)
	200–499	5 (23.8)
	500–999	2 (9.5)
	≥ 1,000	5 (23.8)
Recruitment status	Completed	14 (66.7)
	Recruiting	7 (33.3)
Participating site	Single-center	4 (19.0)
	Hospital	3 (14.3)
	Primary clinic	1 (4.8)
	Multi-center (< 10)	11 (52.4)
	Hospital	10 (47.6)
	Combined	1 (4.8)
	Multi-center (≥ 10)	6 (28.6)
	Hospital	1 (4.8)
	Primary clinic	5 (23.8)
Publication	Yes	10 (47.6)
	No	11 (52.4)

### 3.5 Outcomes measured in KM registries

**Figure 3:**
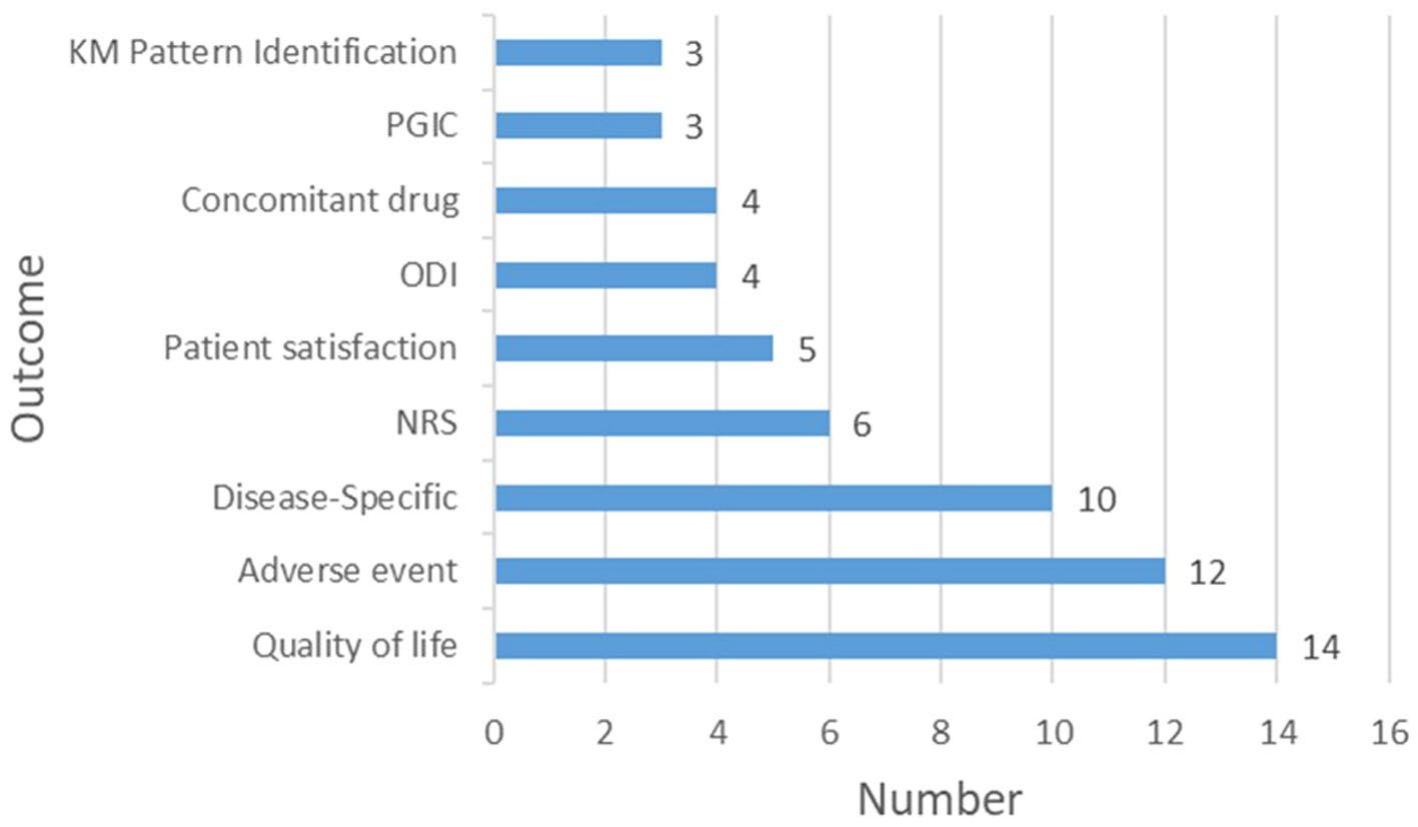
Outcome measures in Korean Medicine registries. KM, Korean Medicine; PGIC, Patient global impression of change; ODI: Oswestry disability index; NRS: Numeric rating scale.

The most frequently assessed outcome was quality of life [14 (66.7%)] registries, underscoring the impact of KM treatments on patient well-being. Subsequently, adverse events were monitored in 12 (57.1%) registries to ensure safety of KM interventions. Disease-specific outcomes, including the Shoulder Pain and Disability Index, Body Mass Index, Total Nasal Symptom Score, the House-Brackmann scale, and stroke evaluation metrics, were tracked in 10 (47.6%) registries to evaluate treatment efficacy across various conditions. Other measured outcomes included the Numeric Rating Scale score for pain [6 (28.6%) registries], patient satisfaction [5 (23.8%) registries], the Oswestry Disability Index for back pain disability and Concomitant Drug use [4 (19.0%) registries], and the Patient Global Impression of Change and KM Pattern Identification [3 (14%) registries each] (Figure 3; Supplementary Table S1).

## 4 Discussion

We conducted a comprehensive review of KM registries listed in ClinicalTrials.gov, the ICTRP, and the CRIS from inception up to the year 2023. The analysis revealed a consistent increase in the number of KM registry studies (one in 2015 to seven in 2023). This upward trajectory highlights the growing interest in registry research within the KM community and illustrates the expanding commitment to documenting and validating the efficacy and safety of KM practices through clinical registries. This trend is especially significant given the unique challenges of conducting large-scale studies in KM clinical settings, which often operate within resource-constrained environments (14, 15). The increase in registry studies serves as an adaptive strategy for comprehensive collection of KM data, which enhances the evidence base within the constraints of traditional clinical settings.

This review of KM registries showed that musculoskeletal disorders were most extensively studied condition. This is consistent with the findings of a 2022 survey indicating that 74.8% of patients sought KM treatments specifically for musculoskeletal issues (16), and thus emphasizes the significant role of KM in addressing these ailments. Furthermore, the diversity in research topics extends beyond musculoskeletal disorders, covering a wide spectrum of health conditions ranging from neurological and respiratory to metabolic diseases (12, 17, 18). This highlights the broad applicability and versatility of KM, showcasing its potential to address diverse health challenges.

Our findings indicated a strong ethical commitment in KM research, with 95.2% of the studies receiving ethics approval. In registry studies, ethics approval is essential for safeguarding participant welfare, ensuring data integrity, and maintaining public trust in the research process (2018) (19). In our review, hospitals were the leading sponsor organizations (61.9%), with universities and research institutions also providing significant support, which highlights the wide-ranging backing for KM research. Moreover, the registries primarily focused on specific diseases or conditions (76.2%), treatment methods (38.1%), and patient demographics (23.8%), which demonstrates the diversity of studies within the KM field. Moreover, majority of the studies were multi-center studies, emphasizing collaboration and comprehensive research. Furthermore, 112 primary clinics and 68 hospitals participated in registries, highlighting the essential role of primary clinics in KM research. This is consistent with the fact that >85% of KM institutions operate as primary care clinics (3, 16), which underscores the extensive involvement of various healthcare environments in KM studies.

In KM registries, quality of life emerged as the primary outcome [14 (66.7%) registries], highlighting the positive effects of KM treatments on well-being. Subsequently, 12 (57.1%) registries tracked adverse events, emphasizing the commitment to the safety of KM practices. This is consistent with previous findings indicating that KM treatments contribute to enhancing the quality of life and are recognized as safe therapeutic options (20–23). Disease-specific outcomes, along with metrics such as pain and patient satisfaction, illustrate a broad approach for evaluating the efficacy of KM. However, the limited inclusion of KM Pattern Identification (only three registries) and the overall scarcity of unique characteristics of KM suggest that the research may not fully capture or utilize the unique aspects associated with KM. This gap could restrict the selection of suitable participants and accurate evaluation of the efficacy of KM, which May lead to overlooking of its holistic and individualized nature. This highlights the necessity for future research to comprehensively integrate and elaborate on the distinct principles and practices of KM.

In this review, only one KM registry study (KCT0006625) published both its protocol and results, indicating a significant transparency gap in KM research (12, 13). This issue impedes identification of publication bias and selective reporting, and thus challenges the integrity and assessment of KM findings (24). It is important to improve publication practices of KM in order to ensure transparency, uphold ethical standards in clinical research, build trust, and maintain the relevance of research findings. Publishing both study protocols and outcomes is key to validating research credibility. Future efforts should aim to broaden the scope of research, improve the sharing of findings, and foster collaborations that will deepen the understanding and application of KM in addressing a wide range of health challenges.

To address these critical issues, we propose several recommendations. Large-scale studies are warranted to comprehensively evaluate the effectiveness and safety of KM treatments, as well as to elucidate underlying KM theories. Given that >85% of KM care is delivered in primary clinics (3, 16), their inclusion in studies is vital for capturing real-world practices. Establishing incentive systems, including certifying clinics that use standardized electronic medical records for registry contributions, can significantly enhance data collection, and thus facilitate comprehensive research representation of KM (25). Furthermore, developing a registry for treatment methods that accommodates the unique diagnostic terminologies of KM, alongside long-term observation of patients, will provide insights into the impact of KM on chronic conditions and quality of life (26, 27). Research should not only focus on data collection but also aim to improve patient care through measurement-based care approaches, offering patients tangible benefits from their participation (28). Finally, the development and widespread adoption of a user-friendly, comprehensive electronic medical record system that is tailored to the specific needs of KM will support retrospective and prospective data collection, and thus ultimately advance the integration of KM into the broader healthcare landscape.

## 5 Conclusion

Our comprehensive review highlights the growth and potential of KM research in South Korea, emphasizing the need for greater transparency and integration of its unique attributes. The increasing number of registry studies signals a shift toward evidence-based practices. However, there remain challenges, including the limited inclusion of unique characteristics of KM and gaps in publishing protocols and results. Addressing these issues is crucial for advancing the credibility and utility of KM in healthcare. Future efforts should focus on enhancing publication standards, deepening research into holistic principles, fostering its role in integrative medicine, and improving patient care.

## Data Availability

The datasets presented in this study can be found in online repositories. The names of the repository/repositories and accession number(s) can be found in the article/Supplementary material.
