# Supplementary material for: Comprehensive review of Korean Medicine registries 2015–2023
Source: Front Med (Lausanne). 2024 Sep 18;11:1412053. doi: 10.3389/fmed.2024.1412053 (PMC11445122; doi:10.3389/fmed.2024.1412053)
Supplement: Supplementary file 1 [file Table_1.DOCX]

Table S1 Overview of Korean Medicine Registry Studies

| **Study Title** | **Year** | **Sponsor Organization** | **Number of Site** | **Sample Size** | **Condition(s)/Problem(s)** | **Primary Outcomes** | **Secondary Outcomes** | **Publication** |
| --- | --- | --- | --- | --- | --- | --- | --- | --- |
| Korean medicine registry for Low back pain-prospective observational multicenter study | 2015 | Gil Korean Medicine Hospital, Gachon University | 7 Hospitals | 150 | (M00-M99)Diseases of the musculoskeletal system and connective tissue | Roland Morris Disability Questionnaire | 1. Pattern Identification Questionnaire of Low Back Pain 2. Characterisitics of Low Back Pain 3. Waist Range of Motion 4. Neurological Examination for Low Back Pain 5. Numeric Rating Scale of Pain (NRS) 6. European Quality of life 5 Dimension (EQ-5D) | Protocol [1] |
| Registry for Korean Medicine and Western Medicine collaborative treatment | 2017 | Pusan National University Korean Medicine Hospital | 12 Hospitals | 1000 | (M54.56)Low back pain, lumbar region " | 1. Analysis the awareness of a demonstration project 2. Analysis the variables of the most frequent diseases in a collaborative treatment  3. Analysis the process and items of a collaborative treatment. 4. Analysis the necessity of a demonstration project and satisfaction with a collaborative treatment.  5. Analysis the necessity of improving the treatment progress and procedures of a demonstration project. 6. Analysis the necessity of improving in the overall aspect of the demonstration project | 1. Demographic variables, such as sex, age, etc. 2. Socioeconomic variables, such as education level, average monthly income per household, etc. 3. Variables of insurance coverage excluding national health insurance. 4. Experience variables of the hospital of a collaborative treatment. | Not published |
| A Construction of Cancer Registry and Prospective Korean Medicinal Cancer Cohort Study in East-West Cancer Center, Dunsan Korean Medicine Hospital of Daejeon University : Focused on Lung, Breast, Stomach, Colorectal, Liver, Uterine and Ovarian Cancers | 2016 | Daejeon Korean Medicine Hospital of Daejeon University | 1 Hospitals | 172 | Not Applicable-Etc | Disease-free survival (DFS) | 1. Overall survival (OS) 2. The Eastern Cooperative Oncology Group Performance Status 3. EORTC QLQ-C30 (quality of life) 4. Beck Depression Inventory-Ⅱ 5. Spielberger&#39;s State-Trait Anxiety Inventory-form Korean YZ 6. Blood test results 7. Telomere length 8. adverse events (Common Terminology Criteria for Adverse Events; CTCAE v4.03) | Protocol [2] |
| Clinical research of Korean medicine and Western medicine Collaboration Registry for Low back pain : A Pilot Study | 2017 | Pusan National University Korean Medicine Hospital | 2 Hospitals | 120 | (C00-D48)Neoplasms | Numeric Rating Scale(NRS) | 1. Collaboration medicine associate information survey 2. Oswestry disability index (ODI) 3. EuroQol-5 Dimension(EQ-5D) 4. Patient global impression of change (PGIC) 5. Diagnostic list, Treatment list | Protocol [3] |
| A prospective observational study of optimal acupoint selection on patients with functional gastrointestinal disorders | 2023 | Kyung Hee University | 1 Hospitals | 420 | (C34.99)Malignant neoplasm of bronchus or lung, unspecified, unspecified side " | 1. Change of scores in questionnaire : Nepean Dyspepsia Index Questionnaire 2. Change of scores in questionnaire : Functional Dyspepsia Quality of Life Questionnaire 3. Change of scores in questionnaire : Irritable Bowel Syndrome Quality of Life Questionnaire | Pattern identification of patients and extracted features : Standard Tool for Pattern Identification of Functional Dyspepsia | Protocol [4] |
| Korean Medicine Cancer Registry (KMCARE) based Prospective Multi-Centered Observational Study | 2022 | Kyung Hee University Oriental Medicine Hospital at Gangdon | 4 Hospitals | 650 | (M00-M99)Diseases of the musculoskeletal system and connective tissue | The Functional Assessment of Cancer Therapy - General (FACT-G) | 1. MDASI-core 2. Body Constitution Questionnaire (BCQ) 3. PFS 4. OS (from enrolled day to event) | Protocol [5] |
| The safety assessment of pharmacopuncture on musculoskeletal patients: a multi-center, registry | 2022 | Jaseng Hospital of Korean Medicine | 7 Hospitals | 1000 | (M54.56)Low back pain, lumbar region " | Incidence of adverse events | Number of abnormal cases that appeared on the blood analysis | Not published |
| A multi center and non interventional registry of Korean Medicine Treatment for Patients with Shoulder pain. | 2022 | Kyung Hee University Oriental Medicine Hospital at Gangdong | 5 Hospitals | 400 | (K00-K93)Diseases of the digestive system | 100mm pain VAS (visual analogue scale) | 1. Shoulder Pain and Disability Index (SPADI)  2. Shoulder range of motion (ROM)  3. EuroQol-5 dimensions-5 Levels (EQ-5D-5L) 4. Treatment satisfaction evaluation 5. Safety - Adverse event | Not published |
| Weight Control Registry using Korean Medicine: A Prospective Registry Study | 2022 | Dongguk University Gyeongju Campus | 4 Hospitals 1 Primary clinics | 200 | (K30)Functional dyspepsia | Weight | 1. Body composition test 2. Liver function test, Renal function test and lipid test 3. Korean medicine pattern identification test 4. Korean version of Obesity-related QOL scale 5. Tongue diagnosis test, Pulse diagnosis test and Face color test 6. Adverse events | Protocol [6] |
| Korean Medicine Registry of Herbal Medicine for Weight Loss | 2021 | Kyung Hee University Oriental Medical Center | 15 Primary clinics | 1000 |  | 1. Body mass index  2. Body composition test  3. Liver and kidney function tests 4. Adverse event | 1. blood pressure 2. pulse rate 3. body temperature | Protocol [7] |
| Combined Korean medicine therapies in children with allergic rhinitis: A multi-center, observational explanatory registry trial | 2021 | Kyung Hee University | 15 Primary clinics | 120 | Functional dyspepsia(FD) and irritable bowel syndrome(IBS)" | 1. Total nasal symptom score, TNSS 2. Numerical rating scales, NRS | 1. Quality of life questionnaire in Korean children with allergic rhinitis 2. PedsQLTM 4.0 Generic Core Scale 3. Paediatric Allergic Disease Quality of Life Questionnaire; PADQLQ | Protocol [8], Results [9] |
| Patient Registry Study on the Clinical Course Observation and Data Collection of Saegmaeksangagambang for Hypertensive Patients | 2023 | Bichedam Korean Medicine Clinic | 1 Primary clinics | 30 | (C00-D48)Neoplasms | Change of mean systolic blood pressure | 1. Change of mean systolic blood pressure 2. Change of mean diastolic blood pressure 3. The rate of normalization of mean sitting systolic/diastolic blood pressure (the rate of patients with mean sitting systolic/diastolic blood pressure below 140/90 mmHg) 4. The rate of Blood pressure responsiveness (the proportion of patients with a reduction in mean sitting systolic blood pressure of 20 mmHg or more, or a reduction in mean sitting diastolic blood pressure of 10 mmHg or more). 5. Changes in individual parameter values of 3D blood pressure waveform analysis equipment. 6. Changes in European Quality of Life Five Dimension Five Level Scale (EQ-5D-5L) questionnaire scores | Not published |
| Safety of Decoction Forms of Herbal Medicine in Postpartum Period: A Registry Study of the Korean Medicine Hospital and Postpartum Care Center | 2023 | Korea Institute of Oriental Medicine | 1 Hospitals | 1000 | (C97)Malignant neoplasms of independent (primary) multiple sites | 1. Adverse event 2. concomitant drug | ECW ratio | Not published |
| Facial Palsy Patients Receiving Korean Medicine Effectiveness Registry | 2023 | Korea Institute of Oriental Medicine | 39 Primary clinics | 413 |  | House-Brackmann scale | 1. EQ-5D, EQ-VAS 2. Korean Medicine Review of System(Digestion, Defecation, Urination, Sleep) 3. When the patient began to perceive improvement 4. Patient satisfaction 5. Adverse events | Not published |
| Stroke Sequelae Patients Receiving Korean Medicine Effectiveness Registry | 2023 | Korea Institute of Oriental Medicine | 28 Primary clinics | 84 | Malignant Neoplasms" | modified MYMOP2 | 1. EQ-5D, EQ-VAS 2. Korean Medicine Review of System(Digestion, Defecation, Urination, Sleep) 3. Patient satisfaction 4. Adverse events | Not published |
| A multicenter registry of neuropsychiatric outpatients in Korean medicine hospitals (KMental): protocol of a prospective, multicenter, registry study | 2021 | Kyung Hee University Oriental Medicine Hospital at Gangdong | 8 Hospitals | 300 | "(M00-M99)Diseases of the musculoskeletal system and connective tissue | Korean-Symptom Check List 95 (KSCL 95) | 1. Concomitant drugs  2. Psychiatric diagnosis  3. Diagnosis of oriental disease  4. Hwabyeong scale-1  5. Hwabyeong scale-2  6. State-Trait Anxiety Inventory-KYZ (STAI-KYZ) 7. State-Trait Anger Expression Inventory (STAXI) 8. Insomnia Severity Index (ISI)  9. Beck Depression Index ll (BDI-ll)  10. Sasang Personality Questionnaire (SPQ)  11. Mibyeong Index (MBI)  12. Self-Assessment from the Perspective of Spectrum Mental Disorders 13. Heart Rate Variability (HRV)  14. 5-Level EQ-5D version 15. Absence of adverse reactions events  16. Disease burden 17. Demographic information | Protocol [10] |
| Registry of Stroke in Korean Medicine Hospital | 2023 | Kyung Hee University Oriental Medical Center | 4 Hospitals | 500 | (M54.9)Dorsalgia, unspecified | 1. emographic information 2. history of stroke, stroke symptoms 3. laboratory tests 4. stroke evaluation (hemiplegia, safety after acupuncture/pharmacopuncture treatment, gait and balance disorders, activity of daily living (ADL) and fatigue, stroke scale, memory and disorientation, depression),  5. Korean medicine treatment details (herbal medicine, acupuncture, electroacupuncture, bee venom injection, pharmacopuncture, indirect moxibustion, direct moxibustion, cupping therapy) 6. rehabilitation treatment details (physical therapy, occupational therapy, speech therapy, swallowing therapy) 7. combination therapy ( Western medicine) | N/R | Not published |
| Clinical effectiveness of herbal medicines for health promotion: a registry in practice-based research networks (PBRN) | 2023 | Daegu Haany University | 13 Primary clinics | 100 |  | Patients' Global Impression of Change (PGIC) | 1. Gastrointestinal Symptom Rating Scale (GSRS)  2. Overactive Bladder Symptom Score Questionnaire (OBSSQ), International Prostate Symptom Score (IPSS) 3. Leeds Sleep Evaluation Questionnaire (LSEQ) 4. Fatigue Severity Scale (FSS) 5. Depression Anxiety Stress Scale 21 (DASS-21)  6. Adverse events | Not published |
| Concomitant treatment of Chuna manual therapy and pharmacopuncture on Neck & Back pain in patients with Traffic Injuries : A preliminary, prospective, observational registry study | 2018 | Pusan National University Korean Medicine Hospital | 3 Hospitals | 120 | Low back pain" | Numeric Rating Scale (NRS) | 1. Disability Index [Oswestry Disability Index (ODI) /Neck Disability Index (NDI)] 2. EuroQol-5 Dimension (EQ-5D)  3. Satisfaction (9-point Likert scale) 4. Adverse events | Not published |
| Korean Medicine Patient Registry of Postoperative Therapy for Musculoskeletal Disorders (KPOP-MD): A Study Protocol | 2019 | Gachon University Gil Oriental Medical Hospital | 5 Hospitals | 150 | "(M00-M99)Diseases of the musculoskeletal system and connective tissue | 1. Assessment of musculoskeletal pain intensity by Numeric Rating Scale (NRS) 2. Disability and estimating quality of life by Oswestry Disability Index (ODI) 3. Disability and estimating quality of life by Oswestry Disability Index 4. Assessment of Pain and disability of the shoulder by SPADI questionnaire 5. The health status will be observed with the Euroqol (EQ-5D-5L) questionnaire. 6. Severity assessment of any symptoms | N/R | Not published |
| Observational Study on Effectiveness and Safety of Integrative Korean Medicine Treatment for Inpatients With Sciatica Due to Lumbar Intervertebral Disc Herniation | 2019 | Jaseng Medical Foundation | 4 Hospitals | 1000 | (M25.5)Pain in joint | 1. Oswestry Disability Index(ODI)  2. Patient global impression of change (PGIC) 3. Numeric rating scale (NRS) of low back pain, and radiating leg pain | 1. EuroQol-5 Dimension(EQ-5D) 2. Lumbar Range of Motion (FLEX/EXT/LAT/ROTATION) 3. Straight Leg Raise test(SLR test) 4. Adverse events | Protocol [11] |

# References

1. Ko, Y., et al., *Korean medicine registry for low back pain - A study protocol for prospective observational multi-center study (KLOS).* Integr Med Res, 2020. **9**(4): p. 100448.

2. Bae, K., et al., *The effectiveness of anticancer traditional Korean medicine treatment on the survival in patients with lung, breast, gastric, colorectal, hepatic, uterine, or ovarian cancer: A prospective cohort study protocol.* Medicine (Baltimore), 2018. **97**(41): p. e12444.

3. Kim, B.-J., et al., *Clinical research of korean medicine and western medicine collaboration registry for low back pain: A pilot study protocol.* Journal of Korean Medicine Rehabilitation, 2017. **27**(3): p. 117-124.

4. Moon, H., et al., *A prospective observational study of optimal acupoint selection on patients with functional gastrointestinal disorders.* Medicine, 2023. **102**(28): p. e34316.

5. Lee, J.Y., et al., *A Prospective Multi-Centered Registry-Based Observational Study for Patients With Cancer: Design and Rationale for Korean Medicine Cancer Registry (KMCARE).* Integrative Cancer Therapies, 2024. **23**: p. 15347354231223496.

6. Cha, J., et al., *Weight Control Registry Using Korean Medicine: A Protocol for a Prospective Registry Study.* International Journal of Environmental Research and Public Health, 2022. **19**(21): p. 13903.

7. Ko, M.M., et al., *Korean medicine registry of herbal medicine for weight loss.* Medicine (Baltimore), 2022. **101**(23): p. e29407.

8. Chu, H., et al., *Combined Korean medicine therapies in children with allergic rhinitis: A multi-center, observational explanatory registry trial: A study protocol.* Medicine (Baltimore), 2021. **100**(51): p. e28181.

9. Chu, H., et al., *Combined Korean medicine therapies in children with allergic rhinitis: A prospective, multicenter, observational registry study at primary-care network clinics.* European Journal of Integrative Medicine, 2024. **66**: p. 102336.

10. Suh, H.W., et al., *A multicenter registry of neuropsychiatric outpatients in Korean medicine hospitals (KMental): Protocol of a prospective, multicenter, registry study.* Medicine (Baltimore), 2022. **101**(49): p. e32151.

11. Lee, Y.J., et al., *Observational study on effectiveness and safety of integrative Korean medicine treatment for inpatients with sciatica due to lumbar intervertebral disc herniation.* Medicine (Baltimore), 2020. **99**(21): p. e20083.
